# Supplementary material for: Therapeutic Drug Monitoring as a Tool for the Clinical Outcome Prediction in Vedolizumab-Treated Patients: An Italian Pilot Study
Source: Biomedicines. 2024 Apr 9;12(4):824. doi: 10.3390/biomedicines12040824 (PMC11048400; doi:10.3390/biomedicines12040824)
Supplement: Supplementary file 1 [file biomedicines-12-00824-s001.zip › biomedicines-2918615-supplementary.pdf]

Table S1. Regression analysis of factors predicting clinical remission at T 12.

	Univariate Analysis			Multivariate Analysis		
	**Odds Ratio**	***p*-Value**	**95% IC**	**Odds Ratio**	***p*-Value**	**95% IC**
Male	1.44	0.57	0.41–5.07			
Age	0.99	0.48	0.95–1.02			
Body Mass Index	1.14	0.18	0.94–1.38			
Active smokers	0.50	0.48	0.07–3.38			
Crohn's disease	1.67	0.43	0.46–6.06			
5–aminosalicylic acid	2.14	0.24	0.59–7.77			
Topical corticosteroids	0.78	0.77	0.16–3.67			
Systemic corticosteroids	0.76	0.67	0.22–2.71			
Immunomodulators	0.80	0.83	0.10–6.32			
HBI T 0	0.94	0.63	0.75–1.20			
HBI T 6	0.65	0.07	0.40–1.04			
PMS T 0	0.59	0.09	0.32–1.09			
PMS T 6	0.47	0.02	0.25–0.89	0.59	0.11	0.31–1.12
C-RP T 0	0.47	0.25	0.13–1.69			
C-RP T 6	0.24	0.04	0.06–0.92	0.36	0.43	0.03–4.59
Hemoglobin T 0	2.14	0.04	1.04–4.43	1.78	0.50	0.33–9.49
Hemoglobin T 6	1.86	0.02	1.11–3.12	1.03	0.95	0.45–2.35
Fecal Calprotectin T 0	0.99	0.25	0.99–1.01			
Fecal Calprotectin T 6	0.99	0.08	0.99–1.00			
Vedolizumab concentrations T 6	1.02	0.03	1.002–1.03	1.01	0.36	0.99–1.04
Ulcerative colitis	1.01	0.80	0.99–1.02			
